# Pneumothorax and Atelectasis Appearing as a Non-Specific Opacity on a Supine Infant’s Chest X-Ray

**DOI:** 10.5334/jbsr.3162

**Published:** 2023-06-30

**Authors:** Thomas Saliba, Grammatina Boitisios

**Affiliations:** 1 ULB, Belgium; 2 Hopital Universitaire Des Enfants Reine Fabiola, Belgium

**Keywords:** Pneumothorax, pediatric, neonatal, esophageal atresia, complication

## Abstract

**Teaching Point:** Thoracic postoperative complications are difficult to diagnose on supine chest X-rays, with pneumothoraxes accompanied by underlying atelectasis presenting as non-specific opacities due to the superposition of the two entities having opposed radiographic characteristics, with one causing lucency and the other opacity.

## Case History

**Figure 1:**
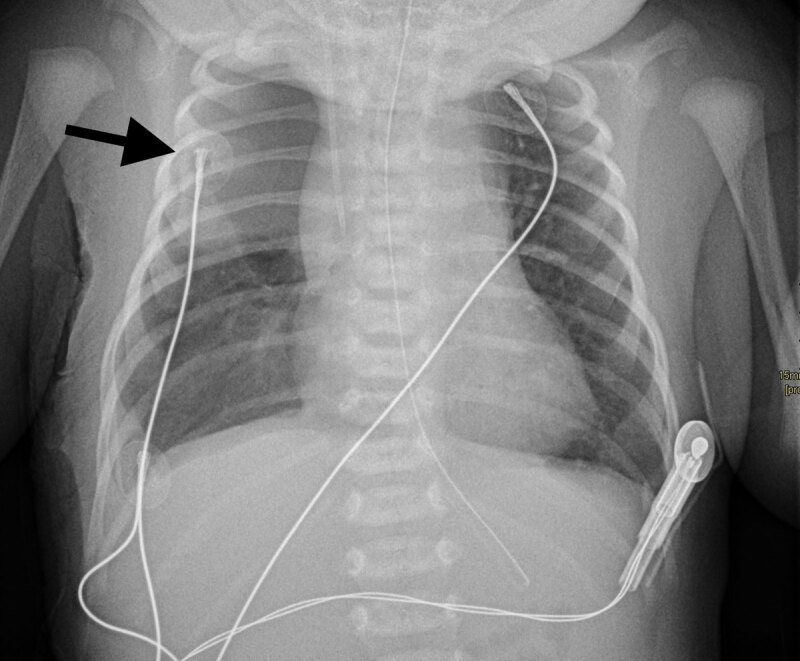


**Figure 2:**
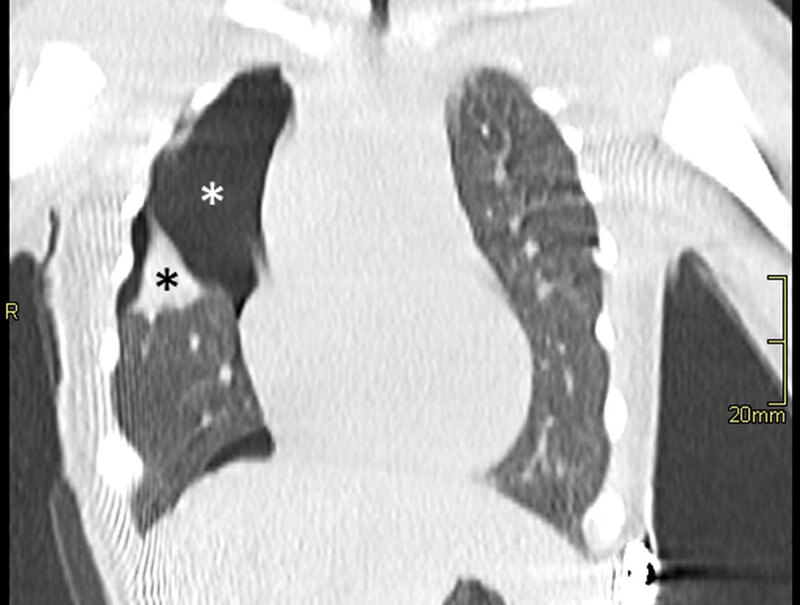


**Figure 3:**
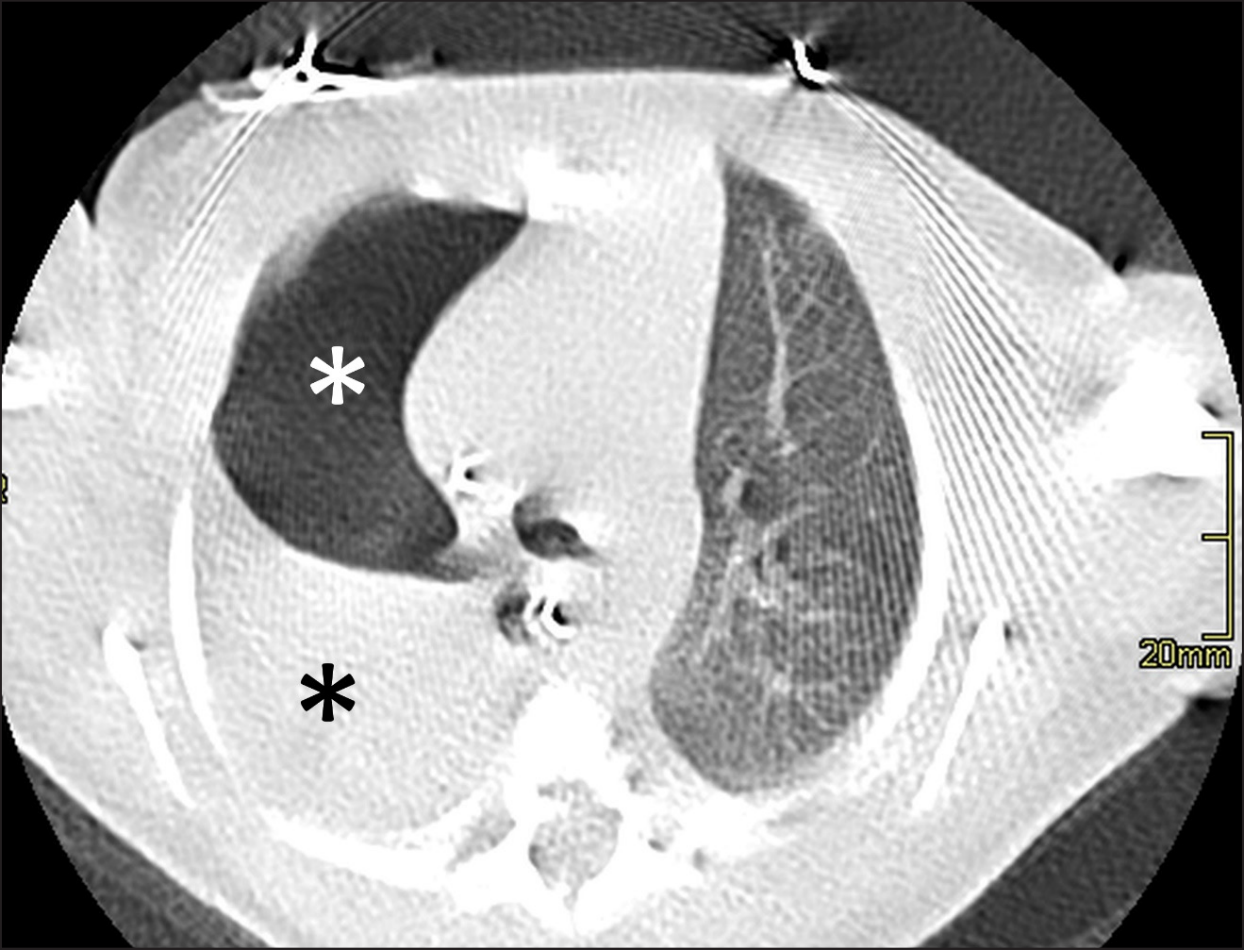


A six-day-old patient, with oesophageal atresia type C, which had been operated on two days prior, with no immediate post-operative complications, presented with sudden desaturation and dyspnoea at 2am, 12 hours after extubation. The paediatrician ordered a supine chest X-ray suspecting a post-operative complication. The supine chest X-ray was performed, revealing a superior right quadrant chest opacity (arrow) (Figure 1). The patient had a correctly positioned central venous line and gastric tube, correctly positioned in the lower left hypogastric region after successful correction of the oesophageal atresia. Pending a radiological opinion, the paediatrician interpreted the exam as a pleural effusion. The radiologist requested a follow-up computed tomography (CT) within the hour to elucidate the origin of the respiratory distress. The CT scan revealed a right sided pneumothorax (white star) with air rising anteriorly and caudally, associated with an underlying lung atelectasis of superior segment of the right lower lobe (black star) on a coronal plane (Figure 2) and axial plane (Figure 3). There was no mediastinal deviation, pleural effusion, or evident cause for the pneumothorax, with occult anastomotic leakage being the presumed cause. The pneumothorax was drained percutaneously, with the patient remaining in the ICU to recover.

## Comments

Oesophageal atresia type C, suffered by our patient, results in a blind ended oesophagus, with the distal oesophagus communicating with the trachea. The incidence of oesophageal atresia with tracheal fistula is around 1/10,000 live births, type C atresia making up 86% of patients [1]. Once a death sentence, most children are now expected to survive, with the life-saving operation occurring with hours of birth [1]. The pathology often presents with other VACTERL (vertebral defects, anal atresia, cardiac defects, tracheoesophageal fistula, renal anomalies, and limb abnormalities), though our patient had none. Despite improved mortality, there are still significant post-operative complications associated with the surgery [1]. These most common short-term complications are anastomosis leakage, tension pneumothorax and sepsis, though longer-term complications also exist such as stricture, fistula recurrence, dysphagia, and oesophageal reflux [1]. Most short-term complications can be suspected on plain chest radiographs; however, the supine position of the patients makes the diagnosis far more challenging. It is important to consider other methods of detecting complications, such as ultrasound, as this population is already heavily burdened by high irradiations due to the amount of follow-up exams. This case demonstrates the difficult nature of diagnosing post-operative complications on plain radiographs, with a non-specific opacity being revealed to be a significant pneumothorax and atelectasis, which may have been seen on a sagittal tangent radiograph, but as the paediatrician was convinced that a pleural effusion was the cause, none was performed.
